# CPR-Centered Redox Interactions Between Cytochrome P450 Oxidoreductase and Cytochrome c Oxidase: Potential Pharmacological Implications

**DOI:** 10.3390/ijms27209075

**Published:** 2026-10-13

**Authors:** Vladimir S. Ponamarev

**Affiliations:** Department of the Pharmacology and Toxicology, Federal State Budgetary Educational Institution of Higher Education “Saint-Petersburg State University of Veterinary Medicine”, 196084 Saint Petersburg, Russia; nir@spbguvm.ru or psevdopyos@mail.ru; Tel.: +7(921)363-00-56

**Keywords:** NADPH, cytochrome P450 oxidoreductase, cytochrome c oxidase, electron transfer, compartmentalization, NADPH shuttles, drug metabolism

## Abstract

The intracellular pool of nicotinamide adenine dinucleotide phosphate (NADPH) is a limited resource for which multiple enzyme systems compete. Among these systems, microsomal NADPH-cytochrome P450 oxidoreductase (CPR, POR) plays a central role in electron transfer to cytochrome P450 enzymes and can also reduce other redox-active electron acceptors *in vitro*. This review analyzes the molecular basis of competition between CPR-mediated cytochrome P450 reduction and cytochrome c reduction *in vitro* and discusses whether this can be extrapolated to an indirect interplay between CPR and cytochrome c oxidase (COX) in intact cells. *In vitro*, reduced cytochrome c (Fe^2+^) acts as a competitive inhibitor of NADPH-dependent cytochrome P450 reduction, apparently by binding to an overlapping site on CPR. However, CPR and COX are physically separated by subcellular compartmentalization, and cytosolic and mitochondrial NADPH pools are largely separate; therefore, CPR and COX should not be considered direct competitors for a common NADPH pool under physiological conditions. Potential pharmacological implications include modulation of drug metabolism, cellular energy homeostasis, and toxicity when both systems are challenged, but these remain hypothetical and require direct flux measurements in intact cells.

## 1. Introduction

NADPH is not merely a cofactor. It is a strategic cellular resource, a reducing equivalent whose value is determined by its scarcity and universality. The pentose phosphate pathway, malic enzyme, and isocitrate dehydrogenase are the main NADPH factories, but dozens of enzymes consume it. Two of them occupy a special position. One is the microsomal electron donor for xenobiotic metabolism. The other is the mitochondrial terminal oxidase driving ATP synthesis. Their indirect connection through redox shuttles and cytochrome c raises the question of whether changes in reducing equivalent availability could influence cellular redox balance. However, cytosolic and mitochondrial NADPH pools are largely separate, and NADPH does not freely cross the inner mitochondrial membrane.

Cytochrome P450 oxidoreductase (CPR, EC 1.6.2.4) is a flavoprotein embedded in the endoplasmic reticulum membrane [1,2]. The molecular mass of the enzyme is about 78 kDa [3,4]. It contains two flavin cofactors (FAD and FMN) and functions as the sole electron donor for all microsomal cytochromes P450 [4,5,6]. The crystal structure of rat liver CPR in complex with NADP^+^ revealed that the aromatic residue Trp-677, which stacks against the isoalloxazine ring of the flavin [4,7], blocks access of nicotinamide to FAD. Without CPR, there is no xenobiotic metabolism, no drug inactivation, no steroid hormone synthesis [8,9].

Cytochrome-c oxidase (COX, complex IV) is the terminal enzyme of the respiratory chain [10,11]. This giant multisubunit complex embedded in the inner mitochondrial membrane catalyzes the four-electron reduction of molecular oxygen to water [12,13]. Electrons reach COX from reduced cytochrome c, which in turn receives them from complex III. COX does not consume NADPH directly. It receives electrons from reduced cytochrome c, which is reduced by complex III. Therefore, COX should not be presented as a direct competitor of POR for NADPH (Figure 1).

At first glance, these two enzymes live in different worlds [2,3]. One in the endoplasmic reticulum, the other in mitochondria. One consumes NADPH directly, the other indirectly through cytochrome c. The endoplasmic reticulum and mitochondria have their own NADPH pools. CPR is anchored on the cytosolic side of the endoplasmic reticulum, while cytochrome c is located in the intermembrane space of the mitochondria, and COX is embedded in the inner membrane [14]. In an intact cell, these proteins are spatially separated, and NADPH does not pass freely through the inner mitochondrial membrane. Therefore, the *in vitro* competition between CPR electron acceptors via cytochrome c cannot be directly extrapolated to physiological conditions without additional evidence.

Cytochrome c is a small hemoprotein that normally shuttles between complex III and complex IV in mitochondria. Yet it is also an excellent substrate for CPR *in vitro* [4]. CPR readily reduces cytochrome c, and this reaction is the basis of the standard assay for CPR activity. Moreover, cytochrome c and P450 occupy the same, or substantially overlapping, interaction site on CPR [15]. Reduced cytochrome c (Fe^2+^) acts as a competitive inhibitor of NADPH-dependent cytochrome P450 reduction [15].

This finding, reported by Davydov and colleagues in 1992, provided the basis for the hypothesis of competition between microsomal and mitochondrial electron acceptors *in vitro* [15]. The authors showed that the dissociation constant of the cytochrome P450 LM2-CPR complex is 3.0 ± 1.5 µM, and this constant does not change in the presence of the substrate benzphetamine [15]. Comparative analysis of amino acid sequences revealed detectable similarity between cytochrome c and cytochrome P450 LM2 at positions 68–87 and 121–145, respectively [15,16].

Thus, in reconstituted *in vitro* systems, CPR can act as a branch point for electron transfer. On one side is cytochrome P450, the main physiological acceptor that enables xenobiotic metabolism. On the other side is cytochrome c, which can intercept electrons and direct them into the respiratory chain.

The redox states of the metal centers in COX and CYP, and of the flavin cofactors in CPR, determine electron transfer efficiency. However, the presence of metal centers in COX and CYP does not imply a direct mechanistic link between CPR and COX [11,17,18].

The potential pharmacological implications of this *in vitro* competition merit careful consideration [7,9]. At the same time, they may affect mitochondrial respiration, altering the cellular demand for NADPH. The question arises: does this create a hypothetical cycle in which activation of one system may indirectly affect the other?

CPR also competes for NADPH with other enzymes, including NADPH oxidases, glutathione reductase, and thioredoxin reductase [3,8,19]. This interaction creates a complex regulatory network in which a change in the activity of one enzyme can have cascading consequences for the entire cell. For example, high microsomal oxidation activity (CPR) can lower the overall cellular NADPH pool, indirectly affecting all NADPH-dependent processes, including maintenance of the reducing potential for mitochondrial chain function [6,20].

Cytochrome b_5_ represents an additional electron transfer pathway. It can receive electrons either from NADH (via cytochrome b_5_ reductase) or from NADPH (via CPR) [3,10,21]. In turn, cytochrome b_5_ can transfer electrons to cytochrome P450, offering an alternative route for the second electron in the P450 catalytic cycle [22,23]. Binding of increasing amounts of purified cytochrome b_5_ to rabbit liver microsomes causes progressive inhibition of CPR activity, accompanied by similar inhibition of NADPH-dependent benzphetamine demethylation [6,20,24]. This indicates that cytochrome b_5_ can compete with cytochrome P450 for electrons from CPR [19,21].

This review was aimed to systematize the known data on the molecular mechanisms of *in vitro* competition between CPR electron acceptors and to discuss the possibility of indirect CPR–COX crosstalk through compartmentalized NADPH pools and cytochrome c. The kinetic parameters of this *in vitro* competition, the structural basis of interactions, and the potential pharmacological implications of this interplay will be discussed. The review also addresses contradictions in the literature, including whether the binding sites for cytochrome c and cytochrome P450 on CPR are truly identical or only partially overlapping. Finally, a conceptual model is proposed as a framework for future studies and hypothesis generation, not as a basis for immediate clinical prediction.

## 2. Materials and Methods

For the preparation of this review, a narrative literature search was conducted in the PubMed, Scopus, and Web of Science databases for the period from 1980 to 2025. The search was carried out between January and March 2025. An updated search was performed in September 2026 to include recent publications. This review is descriptive and does not involve formal quantitative synthesis or meta-analysis. Data are presented as reported in the original publications.

Search queries included combinations of the following keywords and their variants: “NADPH-cytochrome P450 reductase”, “cytochrome P450 oxidoreductase”, “cytochrome c”, “cytochrome c oxidase”, “competitive inhibition”, “electron transfer”, “protein-protein interaction”, “NADPH pool”, “redox regulation”, “flavoprotein”, “heme-copper oxidase”, “FMN domain”, “FAD domain”, “site-directed mutagenesis”, “kinetic mechanism”.

Additionally, references cited in the retrieved reviews and original articles were analyzed to identify relevant publications not indexed in the major databases. Inclusion criteria were original research or reviews, availability of quantitative kinetic or structural data, publication in peer-reviewed journals, and presence of a DOI.

Kinetic parameters of enzymes were obtained from published works that used standard spectrophotometric activity assays. CPR activity was assessed by the rate of cytochrome c reduction at 550 nm in the presence of NADPH. Michaelis constants (K_m_) and inhibition constants (K_i_) were calculated using Michaelis–Menten and competitive inhibition models.

Structural data were derived from studies employing X-ray crystallography, nuclear magnetic resonance (NMR), neutron scattering, and site-directed mutagenesis. For analysis of cytochrome c binding to the FMN-binding domain of CPR, chemical shift mapping in NMR titrations and isothermal titration calorimetry were used [5,25].

To evaluate pharmacological consequences, studies on animal models with conditional POR deletion in various tissues, as well as works using siRNA-mediated suppression of CPR expression in hepatoma cell lines, were analyzed. Additionally, studies on CPR inhibition by various pharmacological agents, including tannic acid and ebselen, were reviewed.

## 3. Results

### 3.1. Kinetic Parameters of In Vitro Competition Between CPR Electron Acceptors

CPR exhibits high affinity for NADPH. For guinea pig liver CPR, K_m_ for NADPH is 3.1 µM, and for cytochrome c it is 26.7 µM. For pig adrenal CPR, K_m_ values for cytochrome c range from 21.3 to 40.8 µM depending on the enzyme isoform. The affinity of CPR for NADPH is 10-fold higher than for NADP^+^: Kd = 0.31 µM for NADPH and 3.3 µM for NADP^+^. This means that at physiological nucleotide concentrations the enzyme is almost completely in the NADPH-bound state. NADP^+^ and 2′-AMP inhibit CPR competitively with respect to NADPH and non-competitively with respect to cytochrome c (Table 1).

The kinetic parameters of CPR vary substantially depending on the source organism, isoform, and experimental conditions [27,28]. For rat liver CPR, the K_m_ for NADPH is 5 µM with either cytochrome P450 or cytochrome c as the electron acceptor, while the K_m_ for cytochrome c is 8 ± 2 µM and for cytochrome P450 is 0.09 ± 0.01 µM, indicating a much higher affinity for the physiological acceptor [29]. Yeast CPR exhibits a K_m_ of 1.59 µM for cytochrome c and 1.46 µM for NADPH, with a V_max_ of 721 pmol/min/pmol enzyme toward cytochrome c. Recombinant Arabidopsis CPR (ATR2) shows a K_m_ of 15 ± 2 µM for cytochrome c and a specific activity of 9.4 µmol/min/mg for cytochrome c reduction [30]. CPR from *Artemisia annua* (aaCPR) displays a ~30-fold weaker apparent binding affinity for the oxidized coenzyme compared to human CPR, a 3.5-fold increase in uncoupled NADPH oxidation, and an 85–100-fold preference for NADPH over NADH [31]. These species-specific differences in cofactor binding, uncoupling efficiency, and acceptor preference highlight the difficulty of extrapolating kinetic data between organisms and experimental systems.

The kinetic mechanism of CPR has been characterized as a random Bi-Bi mechanism with the formation of a ternary CPR-NADPH-electron acceptor complex as the catalytic intermediate. Reduced cytochrome c does not affect enzyme activity, indicating that the product does not inhibit the enzyme by feedback. Kinetic studies using mutant forms of CPR showed that replacement of Asp-634 with alanine or asparagine leads to 36-fold and 10-fold reductions in cytochrome c reductase activity, respectively, associated with impaired hydride transfer and slowed NADP^+^ release [18,22].

Steady-state kinetic studies of yeast CPR revealed a random Bi-Bi mechanism with NADPH donating electrons directly to CPR to form a reduced intermediate [30,31]. Stopped-flow analysis of human CPR demonstrated multi-phasic reduction of the flavin absorbance spectra following rapid mixing with NADPH, with FAD reduction occurring without direct spectral evidence for charge-transfer species. In plant CPRs (aaCPR and ATR2), NADPH-dependent FAD reduction is ~50-fold faster than in human CPR, whereas interflavin electron transfer is slower than NADPH hydride transfer, occurring with an observed rate constant of ~50 s^−1^ [30]. These differences in rate-determining steps indicate that the kinetic mechanism of CPR is not uniform across species and may influence the efficiency of electron transfer to competing acceptors.

The key discovery underlying the concept of *in vitro* competition was made by studying the kinetics of NADPH-dependent reduction of cytochrome P450 LM2 in a monomeric reconstituted system. In the absence of substrate, the reduction of cytochrome P450 LM2 proceeds as a monophasic process. Reduced cytochrome c (Fe^2+^) acts as a competitive inhibitor of this reduction [15]. In the presence of 1 mM benzphetamine, an additional extremely fast phase is observed. Under these conditions, reduced cytochrome c was found to be a competitive inhibitor of the slow phase of the reduction process, which accounts for 80% of the total reduction amplitude [15,19].

Inhibition experiments allowed determination of the dissociation constant of the cytochrome P450 LM2–CPR complex: 3.0 ± 1.5 µM [15] (Table 2). This constant was the same in both the presence and absence of benzphetamine [15]. On this basis, the authors proposed that cytochrome P450 and cytochrome c bind to the same or substantially overlapping site on CPR. However, competitive inhibition alone does not prove identical physical binding sites; it may also reflect overlapping, mutually exclusive, or allosterically coupled interactions [15]. Comparative amino acid sequence analysis revealed detectable similarity between cytochrome c and cytochrome P450 LM2, as well as between fragments of CPR and cytochrome b_5_ [16,21].

### 3.2. Structural Aspects of CPR Binding

CPR consists of several domains, among which the FMN-binding domain (FBD) is the direct electron donor to cytochrome c [32]. Electron transfer from FBD to cytochrome c occurs at different rates depending on the redox states of FBD. Compared with full-length CPR, FBD reduces cytochrome c at a higher rate in both the semiquinone and hydroquinone states [10,21,25].

NMR titrations revealed the formation of dynamic complexes between FBD and cytochrome c in fast exchange [33]. Chemical shift mapping identified FBD residues involved in the binding interface with cytochrome c. Most of these residues are located near the solvent-accessible edge of the FMN cofactor and are distributed over the FBD surface.

The structural model of the FBD–cytochrome c complex indicates two possible orientations of complex formation. The predominant complex structure shows the formation of a salt bridge between Glu-213/Glu-214 of FBD and Lys-87 of cytochrome c, which may be necessary for complex formation, and a predicted electron transfer pathway mediated by Lys-13 of cytochrome c (Table 3).

The putative docking surface of CPR with its electron transfer partners has been mapped using X-ray crystallography and electrostatic potential calculations. Cytochrome c (or cytochrome P450) binds “in front of” the CPR molecule, allowing NADPH to enter and exit freely from above, while FMN and FAD are buried in the complex. The bowl-shaped surface near the FMN-binding site is likely the docking site for cytochrome c and the physiological redox partners, including cytochromes P450 and b_5_ [27,34]. The interactions between CPR and cytochrome c are primarily electrostatic in nature, with the putative binding surface of CPR indicated by a distinct positively charged region. These structural data, combined with NMR chemical shift mapping and site-directed mutagenesis, support a model in which the binding sites for cytochrome c and cytochrome P450 overlap but are not completely identical, allowing for differential regulation of electron transfer to different acceptors.

The solution structure of the CPR–cytochrome c complex, determined by neutron scattering, showed that the protein–protein interface includes residues from both the FMN- and FAD-binding domains of CPR [5,32,33]. FMN is located close to the heme of cytochrome c, but distant from FAD.

Despite the conclusion of binding to a single center, there are data indicating differences. Cytochrome c is thought to interact with CPR through a separate, though similar, binding site and in a different manner than cytochromes P450 [25]. Mutations in the cofactor-binding site of CPR can differentially affect activity toward cytochrome c and cytochrome P450. In the work of Strohmaier et al. (2019), it was shown that CPR mutants capable of more efficiently using NADH for cytochrome c reduction do not support P450 activity with NADH [33]. This indicates that the binding sites and electron transfer mechanisms for different acceptors are not fully identical.

N-terminal truncation of the membrane anchor of CPR significantly reduces the ability to support P450 activity but has a more subtle effect on cytochrome c reduction. These effects are attributed to loss of membrane integration [13,24].

Thus, there is a contradiction in the literature. On one hand, kinetic data clearly indicate competitive inhibition and binding to a single site [15]. On the other hand, structural and mutagenesis studies reveal differences in the details of interaction [35]. It is likely that the binding sites are overlapping but not completely identical, which allows for fine regulation.

### 3.3. Compartmentalization and NADPH Pools

CPR and COX are located in different cellular compartments: CPR in the endoplasmic reticulum and COX in mitochondria. NADPH does not freely diffuse across the inner mitochondrial membrane. Cytosolic and mitochondrial NADP(H) pools are largely separate and connected via redox shuttles such as the malate–aspartate and isocitrate–α-ketoglutarate shuttles [14]. Therefore, CPR cannot directly consume mitochondrial NADPH; any influence on mitochondrial respiration would be indirect. Nevertheless, at the whole-cell level, both processes are major consumers of reducing power. High microsomal oxidation activity (CPR) can lower the overall cellular NADPH pool, indirectly affecting all NADPH-dependent processes, including maintenance of the reducing potential for mitochondrial chain function. COX does not consume NADPH directly. It receives electrons from reduced cytochrome c, which is reduced by complex III. Therefore, COX should not be presented as a direct competitor of POR for NADPH. Any influence of CPR activity on mitochondrial respiration would be indirect, mediated by changes in cytosolic NADPH availability and redox shuttles. The capacity of these shuttles is limited, and the two NADPH pools are not freely interchangeable.

CPR also competes for NADPH with other enzymes, including NADPH oxidases, glutathione reductase, and thioredoxin reductase [3,8]. This interaction creates a complex regulatory network in which a change in the activity of one enzyme can have cascading consequences for the entire cell.

CPR, localized in the endoplasmic reticulum membrane, accepts electrons from NADPH and transfers them via FAD and FMN either to cytochrome P450 (main pathway leading to xenobiotic metabolism) or to cytochrome c (alternative pathway) [5]. Reduced cytochrome c can be intercepted by cytochrome-c oxidase in mitochondria, creating competition for reducing equivalents between the two systems.

Figure 2 shows Lineweaver–Burk plots for cytochrome P450 reduction in the absence and presence of increasing concentrations of reduced cytochrome c. The plots demonstrate competitive inhibition: all lines intersect at a single point on the ordinate (1/V_max_), while the slopes increase with rising inhibitor concentration, indicating an increase in apparent K_m_. The K_i_ value calculated from the secondary plot of slope versus inhibitor concentration is 3.0 ± 1.5 µM, corresponding to the dissociation constant of the cytochrome P450 LM2–CPR complex [15,16].

Figure 3 presents an integrative scheme showing the main pathways of NADPH generation and consumption in the cell. The pentose phosphate pathway, malic enzyme, and isocitrate dehydrogenase are the main sources of NADPH. Major consumers include CPR (microsomal xenobiotic oxidation), glutathione reductase (maintenance of reduced glutathione), thioredoxin reductase (maintenance of reduced thioredoxin), NADPH oxidases (production of reactive oxygen species), and the indirect influence on COX via cytochrome c [3,10,11]. NADPH is not transported across intracellular membranes, and multistep shuttles involving compartmentalized redox reactions are used to transfer electrons between the mitochondria and cytosol. Mitochondrial NADPH can be exported to the cytosol via citrate, which is converted to malate, or through the malate-aspartate shuttle. The isocitrate/α-ketoglutarate shuttle couples mitochondrial IDH to cytosolic NADPH production. These shuttle systems operate at limited capacity and do not equilibrate the two NADPH pools, meaning that changes in cytosolic NADPH consumption (e.g., by CPR) are unlikely to be directly compensated by mitochondrial NADPH production without metabolic remodeling [34].

Quantitative flux analysis using deuterium-labeled nutrients and mass spectrometry has enabled the resolution of compartmentalized NADPH metabolism in intact cells [36,37]. The principal cytosolic sources of NADPH are the oxidative pentose phosphate pathway, malic enzyme, and isocitrate dehydrogenase, whereas mitochondrial NADPH is produced mainly by isocitrate dehydrogenase, malic enzyme, and nicotinamide nucleotide transhydrogenase [36,37,38]. In cancer cells, the pentose phosphate pathway is typically the largest cytosolic NADPH source, but the collective contribution of alternative pathways (malic enzyme, isocitrate dehydrogenase, and folate metabolism) is often larger than that of the pentose phosphate pathway alone [37,39]. In differentiating adipocytes, most NADPH is produced by malic enzyme [36]. The relative contributions of these pathways vary with cell type, nutrient availability, and hypoxia, indicating that NADPH production is context-dependent [36,38].

The total cellular NADPH pool is small relative to the flux through pathways that utilize this cofactor, meaning that interconversion between oxidized and reduced states must be tightly coupled across all reactions. In cancer cells, the combined flux through malic enzyme (ME1/ME2) can reach 4.14 µmol/mg/h, with the pentose phosphate pathway typically contributing the largest single share of cytosolic NADPH [36,38]. In some cell types, a basal malic enzyme flux of 6 mM/h has been observed, increasing by 12–14 mM/h in G6PD-deleted clones, indicating compensatory upregulation of alternative NADPH sources. The oxidative pentose phosphate pathway accounts for 80.7% of NADPH production in some genome-scale metabolic models, while isocitrate dehydrogenase contributes 13.4% and purine metabolism 5.9% [38,39]. These quantitative data demonstrate that NADPH production is distributed across multiple pathways and that the contribution of any single pathway is context-dependent. The fraction of cytosolic NADPH consumed by CPR and other microsomal oxidoreductases, and the extent to which changes in CPR activity alter mitochondrial NADPH availability, have not been quantified. Therefore, the proposed competition between CPR and COX should be treated as a hypothesis requiring direct flux measurements in intact cells [36,37].

### 3.4. Metabolic and Pharmacological Significance

The potential pharmacological implications of the *in vitro* competition between CPR electron acceptors may manifest in several aspects [11]. First, many drugs are metabolized by cytochromes P450 and can alter electron flux through CPR [2,9]. This may hypothetically affect NADPH availability for other processes, including mitochondrial respiration, but this remains to be directly demonstrated. Second, some agents can directly affect CPR activity. For example, tannic acid inhibits CPR as measured by cytochrome c reduction [40]. Ebselen at micromolar concentrations markedly inhibits the flow of reducing equivalents from CPR to both cytochrome P450 and cytochrome c [41]. Third, genetic variations in CPR can affect drug and steroid metabolism in humans. Mice with conditional Por deletion in specific tissues or with partial suppression of CPR expression show alterations in xenobiotic metabolism and lipid homeostasis [8].

CPR from Candida tropicalis supports reduction of cytochrome c and ferricyanide with approximately 30% uncoupling during reactions with cytochrome c [42]. This means that not all electrons transferred from NADPH are used efficiently some are wasted, which may be relevant for cellular energy balance. CPR from *Artemisia annua* shows a 3.5-fold increase in uncoupled NADPH oxidation compared with human CPR and a strong preference (85- to 100-fold) for NADPH over NADH [31]. This correlation demonstrates species differences in NADPH utilization efficiency, which may have implications for pharmacology.

## 4. Discussion

The presented data indicate a complex and multifaceted relationship between CPR and COX. The interplay between NADPH-dependent redox processes and cytochrome c may contribute to the cellular balance of reducing equivalents supporting microsomal xenobiotic metabolism and mitochondrial energy metabolism.

Quantitative flux analysis of NADPH metabolism in mammalian cells indicates that the principal cytosolic sources are the pentose phosphate pathway, malic enzyme, and isocitrate dehydrogenase, whereas mitochondrial NADPH is produced mainly by IDH2, malic enzyme, and nicotinamide nucleotide transhydrogenase (NNT) [36,37]. These pools are compartmentalized and connected by redox shuttles rather than by free diffusion of NADPH. The fraction of cytosolic NADPH consumed by CPR and other microsomal oxidoreductases, and the extent to which changes in CPR activity alter mitochondrial NADPH availability, have not been quantified. Therefore, the proposed competition between CPR and COX should be treated as a hypothesis requiring direct flux measurements.

The kinetic parameters of *in vitro* competition suggest that at physiological concentrations of NADPH and cytochrome c, CPR will preferentially direct electrons to cytochrome P450 [3,19,43]. In intact cells, cytochrome c is confined to the mitochondrial intermembrane space and is not accessible to ER-localized CPR. A shift toward cytochrome c reduction by CPR would require release of cytochrome c into the cytosol, which typically occurs during apoptosis and is associated with loss of respiratory chain function, not enhanced COX activity [27,28]. Thus, this scenario should be considered a hypothetical pathological interaction.

The kinetic constants compiled in Table 1 and Table 2 were obtained by different methods, including steady-state spectrophotometric assays, stopped-flow spectroscopy, isothermal titration calorimetry, and NMR titration. Steady-state assays of cytochrome c reduction by human CPR reveal a simple hyperbolic relationship on NADPH concentration, whereas stopped-flow studies of rabbit hepatic CPR demonstrated three phases of reduction: a fast first phase yielding an equilibrium mixture of approximately 70% (FMNH_2_, FAD) and 30% disemiquinone (FMNH, FADH) [27,34]. Isothermal titration calorimetry has been used to measure coenzyme binding to human CPR and its isolated FAD-binding domain, providing direct thermodynamic parameters that are not accessible from steady-state kinetics. NMR titration experiments reveal the formation of dynamic complexes between the FMN-binding domain of CPR and cytochrome c on a fast exchange time scale. These methodological differences limit direct comparison of reported constants: K_m_ values depend on the electron acceptor used, and K_i_ values derived from competitive inhibition should not be equated with the dissociation constant (K_d_) of a protein–protein complex [34]. In particular, competitive inhibition may reflect overlapping, mutually exclusive, or allosterically coupled interactions rather than binding to identical physical sites.

The contradiction between data on a single binding center and data on differences in binding sites for cytochrome c and cytochrome P450 may be resolved by assuming overlapping but not identical sites [15,25,34]. This allows for allosteric regulation: binding of one acceptor may modulate the affinity of CPR for the other. Such a model is consistent with the observation that mutations in different regions of CPR differentially affect activity toward cytochrome c and cytochrome P450.

Both COX and CYP contain metal centers, and CPR contains flavin cofactors. Redox transitions at these centers govern electron transfer, but this does not establish a direct functional link between CPR and COX [12]. Cytochrome P450 contains heme iron, and CPR contains flavin cofactors [4]. The redox state of these metallocenters determines the efficiency of electron transfer and, consequently, the competition for NADPH [15]. This creates a feedback mechanism in which accumulation of reduced cytochrome c suppresses further reduction through CPR, directing electrons into the mitochondrial chain.

The potential pharmacological implications of this *in vitro* competition require further study. Many drugs can influence the balance between the two systems, which may hypothetically lead to unforeseen effects. For example, inhibition of CPR may reduce drug metabolism, but whether this increases mitochondrial NADPH availability is unknown. Activation of CPR may decrease cytosolic NADPH, but the consequences for mitochondrial function remain speculative.

Mouse models with conditional Por deletion provide valuable tools for studying these effects *in vivo* [8]. Further research should aim at quantitative assessment of the contribution of each pathway to the overall NADPH flux in various tissues and under different physiological and pathological conditions.

It is crucial to understand that NADPH consumption extends beyond just CPR and COX. It is crucial to understand that NADPH consumption extends beyond just CPR and COX. NADPH oxidases, along with glutathione reductase and thioredoxin reductase, are also important consumers of NADPH [3,8,11]. This creates a complex regulatory network in which a change in the activity of one enzyme can have cascading consequences for the entire cell. For example, activation of NADPH oxidases during inflammation can deplete the NADPH pool, reducing NADPH availability for CPR and thereby slowing xenobiotic metabolism.

There are also species differences in NADPH utilization efficiency. CPR from Candida tropicalis displays about 30% uncoupling during reactions with cytochrome c, whereas CPR from Artemisia annua shows a 3.5-fold increase in uncoupled NADPH oxidation. These differences may be relevant for pharmacology because the efficiency of NADPH utilization influences the rate of drug metabolism and cellular energy balance.

## 5. Conclusions

Cytochrome P450 oxidoreductase and cytochrome-c oxidase occupy different cellular compartments and participate in distinct redox processes. The best-characterized interaction is the *in vitro* competition between CPR-mediated cytochrome P450 reduction and cytochrome c reduction, in which reduced cytochrome c acts as a competitive inhibitor at an overlapping site on CPR. Whether this *in vitro* competition translates into a physiologically relevant indirect interplay between CPR and COX in intact cells remains hypothetical. Structural studies suggest overlapping but not identical binding sites for different CPR acceptors. Potential pharmacological implications may include modulation of drug metabolism, changes in cellular energy homeostasis, and toxicity under simultaneous load on both systems, but these require experimental validation. Understanding these mechanisms may open avenues for future research and hypothesis generation regarding drug interactions and pharmacological correction strategies. Further research should quantify NADPH flux through each system under various conditions, identify additional regulatory mechanisms, and develop pharmacological tools to modulate this interplay. From a clinical perspective, current evidence is insufficient to recommend dose adjustments. The possibility that patients with obesity or diabetes, who often exhibit mitochondrial dysfunction, may have an altered balance between microsomal oxidation and mitochondrial respiration remains a hypothesis for future testing.

## Figures and Tables

**Figure 1:**
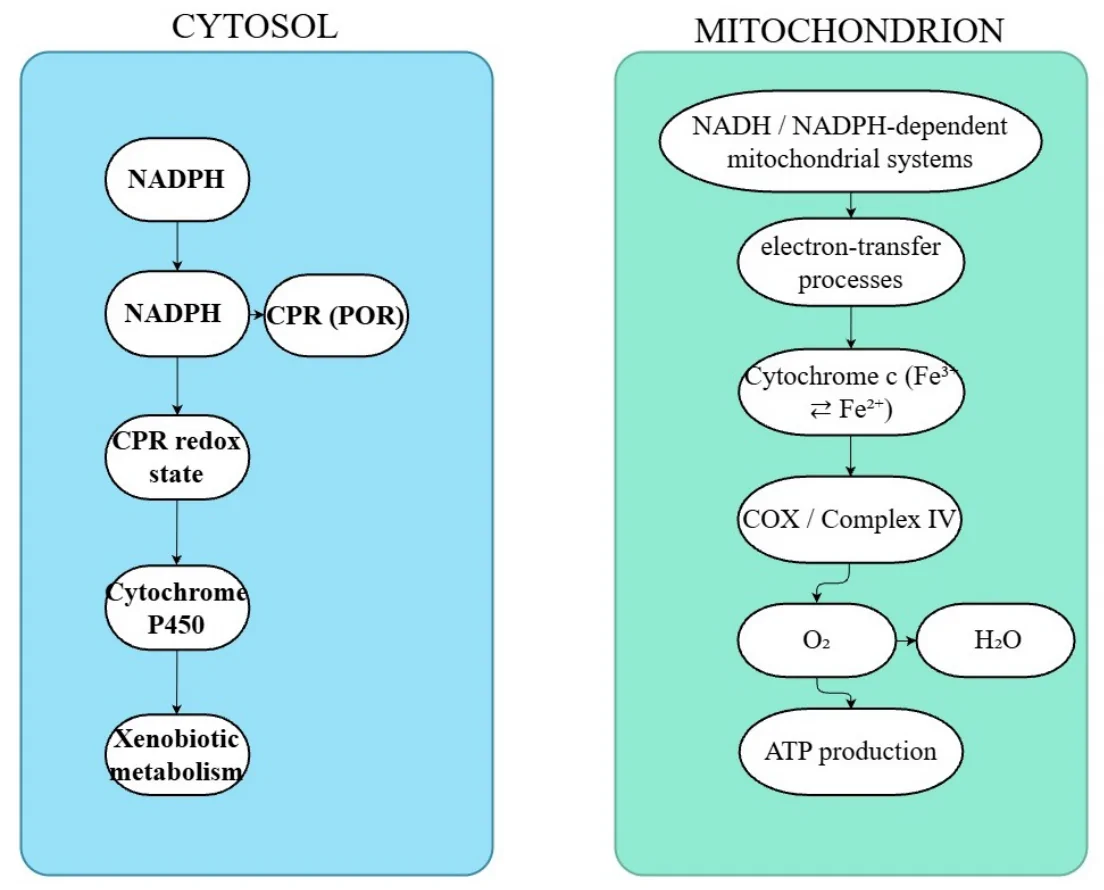
Compartmentalized NADPH metabolism.

**Figure 2:**
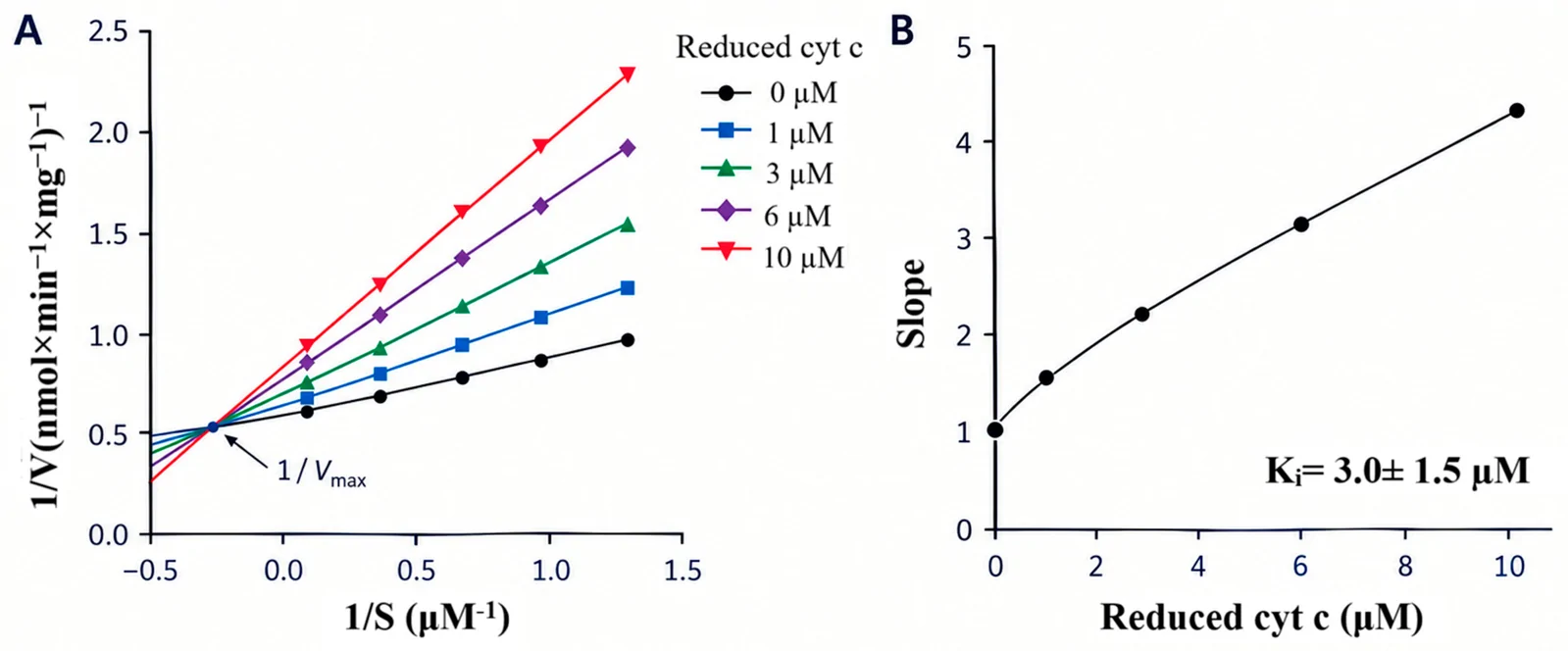
Lineweaver–Burk plots simulated using 1/V = (K_m_/V_max_)(1/S)(1 + [I]/K_i_) + 1/V_max_ with K_i_ = 3.0 µM, based on data from Davydov et al. [15]. (**A**) Lineweaver-Burk plots of cytochrome P450 reduction at increasing concentrations of reduced cytochrome c (0, 1, 3, 6, and 10 µM). (**B**) Secondary plot of the slope versus reduced cytochrome c concentration used to determine the inhibition constant.

**Figure 3:**
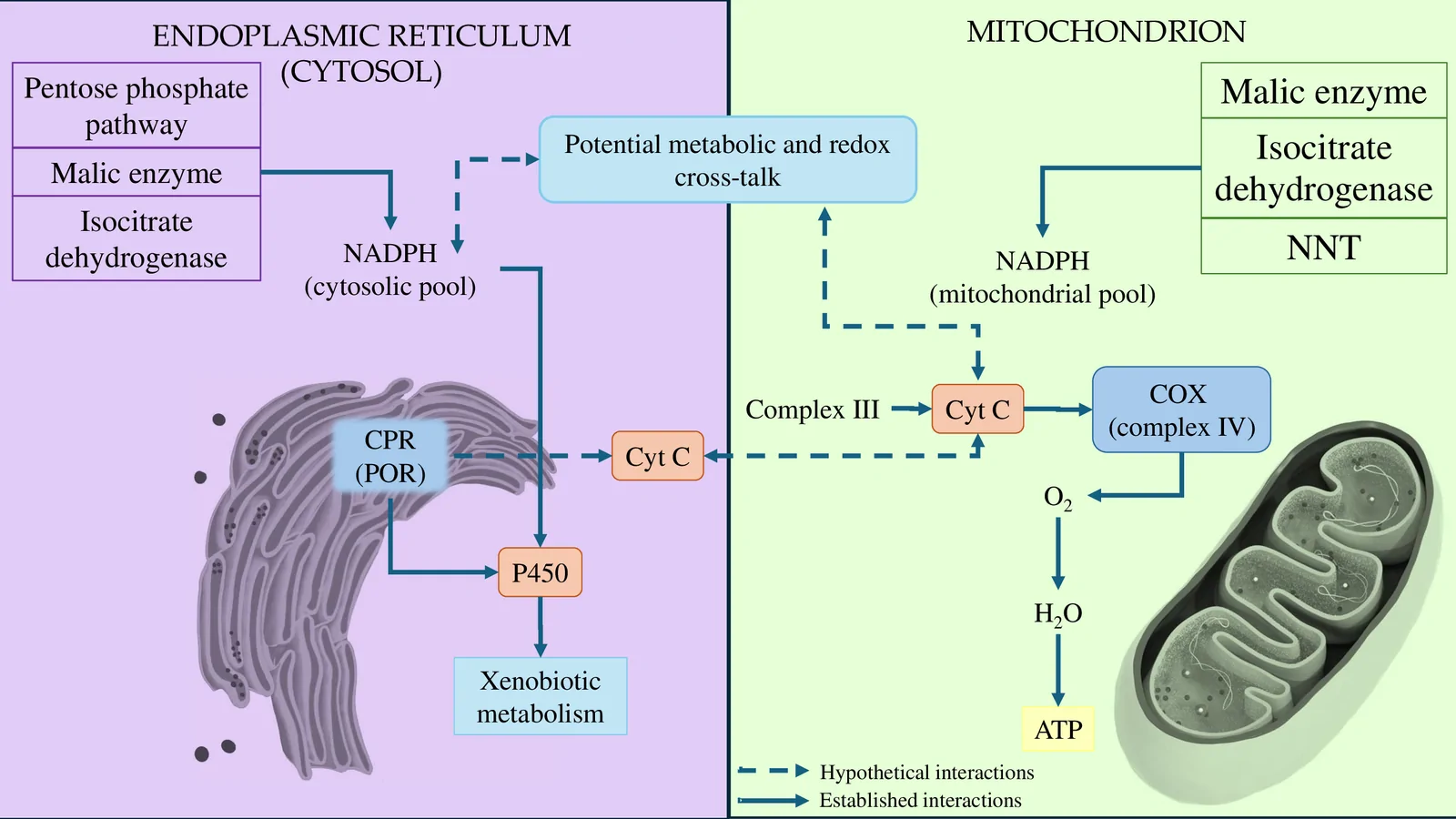
Cellular localization and interplay of redox systems involving CPR, cytochrome P450, and cytochrome c oxidase. Arrow thickness is qualitative and does not imply quantitative flux.

**Table 1:** Kinetic parameters of CPR from different sources.

Source of CPR	Enzyme Form/Construct	Membrane	T, °C	pH	Electron Acceptor	K_m_ (NADPH), µM	K_m_ (Cytochrome c), µM	Kd (NADPH), µM	Reference
Guinea pig liver	Microsomal CPR	Microsomes	25–30	8.0	Cytochrome c	3.1	26.7	-	[4]
Pig adrenal (reductase I)	Microsomal CPR	Microsomes	25–30	7.4–8.0		35.7	40.8	-	[4]
Pig adrenal (reductase II)	Microsomal CPR	Microsomes	25–30	7.4–8.0		22.7	21.3	-	[4]
House fly (recombinant)	Recombinant CPR (purified)	Solubilized	25	7.4		-	-	0.31 (NADPH)	[26]

**Table 2:** Competitive inhibition parameters of cytochrome P450 LM2 reduction by reduced cytochrome c.

Condition	Inhibition Type	Ki (cyt c), µM	Kd (P450-CPR Complex), µM	Fraction of Slow Phase, %	Reference
Without substrate	Competitive	3.0 ± 1.5	3.0 ± 1.5	100	[15,16]
With 1 mM benzphetamine	Competitive (slow phase)	3.0 ± 1.5	3.0 ± 1.5	80	[15,16]

**Table 3:** Key residues involved in the interaction of CPR with cytochrome c.

Protein	Residues	Evidence Type	Role	Reference
CPR (FBD)	Glu-213, Glu-214	crystal structure/NMR	Salt bridge with Lys-87 of cytochrome c.	[34]
Cytochrome c	Lys-87	crystal structure/NMR	Salt bridge with Glu-213/Glu-214 of CPR.	[34]
Cytochrome c	Lys-13	predicted	Proposed electron transfer pathway.	[10,34]
CPR	Asp-634	mutagenesis	Cofactor binding; mutation reduces activity.	[18]

## Data Availability

No new data were created or analyzed in this study. Data sharing is not applicable to this article.

## Abbreviations

The following abbreviations are used in this manuscript:

CYP	cytochrome P450
NADPH	nicotinamide adenine dinucleotide phosphate
CPR, POR	NADPH-cytochrome P450 reductase
FAD	flavin adenine dinucleotide
FMN	flavin mononucleotide
COX, complex IV	cytochrome-c oxidase
ATP	adenosine triphosphate
NMR	nuclear magnetic resonance
FBD	FMN-binding domain
